# Cross-Sectional Study of Polybrominated Flame Retardants and Self-Reported Attention Deficit Hyperactivity Disorder in US Youth Aged 12–15 (NHANES 2003-2004)

**DOI:** 10.1155/2016/2392045

**Published:** 2016-07-04

**Authors:** Jennifer Przybyla, Molly L. Kile, Ellen Smit, E. Andres Houseman

**Affiliations:** School of Biological and Population Health, College of Public Health and Human Sciences, Oregon State University, Corvallis, OR 97330, USA

## Abstract

*Background.* Animal toxicity tests and epidemiological studies suggest that exposure to PBDEs can alter attention behavior, yet few studies have examined their association with diagnosis of attention deficit hyperactivity disorder (ADHD) in adolescents.* Methods.* Logistic regression was used to examine the cross-sectional association between ADHD and lipid and non-lipid adjusted blood serum concentrations of 2′,4-tribromodiphenyl ether (BDE-28), 2,2′,4,4′-tetrabromodiphenylether (BDE-47), 2,2′,4,4′,5-pentabromodiphenyl ether (BDE-99), 2,2′,4,4′,5,5′-pentabromodiphenyl ether (BDE-100), 2,2′,4,4′,5,5′-hexabromodiphenyl ether (BDE-153), serum PBDEs, above/below the 75th percentile of serum PBDEs, and tertiles of serum PBDE in 12–15-year-olds (*N* = 292) using the National Health and Nutrition Examination Survey (NHANES) 2003-2004.* Results.* The ADHD weighted prevalence was 13.57%. The weighted adjusted odds ratios (AOR) and 95% confidence interval (CI) between ADHD diagnosis and lipid adjusted BDE-28, BDE-47, BDE-99, BDE-100, BDE-153, serum total PBDE, serum PBDE concentrations above the 75th percentile, and serum PBDE concentrations in the second or third tertile were 1.16 (95% CI: 0.51, 2.67), 1.36 (95% CI: 0.72, 2.56), 1.51 (95% CI: 0.70, 3.25), 1.53 (95% CI: 0.73, 3.23), 1.43 (95% CI: 0.57, 3.56), 1.41 (0.71, 2.83), 0.59 (0.10, 3.56), 6.16 (1.19, 31.90), and 0.99 (0.23, 4.29).* Conclusions.* We observed no association between serum PBDE concentrations and ADHD in US youths.

## 1. Background

Attention deficit hyperactivity disorder (ADHD) is a common neurodevelopmental disorder in children characterized by forgetfulness, excessive daydreaming, making careless mistakes, and excessive fidgeting [1]. In 2011, 11% of children aged 4–17 were diagnosed with ADHD which represents a 5% increase from 2003 [2]. Children diagnosed with ADHD are more likely to have major nonfatal injuries [3], have a more difficult time with peer relationships, and are more likely to drop out of school [4]. It is estimated that the societal cost of ADHD is between $36 and $52 billion dollars a year, stemming from medical cost and loss of productivity [5].

While the etiology of ADHD is unknown, both environmental and genetic factors are thought to contribute to the development of ADHD [6]. Specifically, environmental contaminants including lead and environmental tobacco smoke have been associated with increased risk of ADHD [7, 8]. Additionally, polybrominated flame retardants which are suspected neurotoxicants [9] and are present in the blood serum of 97% of US citizens [10] have been associated with increased risk of poor attention behavior. A Dutch study observed that maternal blood levels of PBDEs measured in the 35th week of pregnancy were associated with decrease in sustained attention, lower fine manipulative abilities, better coordination and behavior, and better visual perception in 5-6-year-olds [11]. Another large prospective study conducted in the United States also observed that children born to mothers with the highest total serum PBDE levels were more likely to have the highest scores on the ADHD Confidence Index test which indicates a high probability of correct clinical ADHD classification at 5 years of age compared to children born to mothers with the lowest total serum PBDE levels. This study also observed that the sum of maternal serum PBDE exposures was positively associated with Conners' ADHD Diagnostic and Statistical Manual of Mental Disorders, 4th edition (DSM-IV), index scale, whose scores are relevant to clinic diagnosis to ADHD, at 7 years of age [12].

The studies provide some evidence that prenatal exposure to PBDEs is related to ADHD-related behaviors and inattentive behaviors in young children. Yet little is known about the relationship between PBDE exposure and attention and hyperactivity levels in older children. Furthermore, data from experimental studies in rodents exposed to PBDEs during development showed that memory and learning alterations worsened with age, indicating that, even after a delay in exposure, PBDEs may have a lasting effect [13, 14]. Given the ubiquity of PBDEs exposures in the US population and the significant impact of neurodevelopmental disorders on our society, it is important to improve our understanding of the relationship between PBDE exposure and ADHD in older children. Subsequently, we conducted a cross-sectional study examining the association between PBDE serum concentrations and ADHD prevalence in youths aged 12–15 years living in the US using data collected by the National Health and Nutrition Examination Survey (NHANES).

## 2. Methods

### 2.1. Study Population

This cross-sectional study used data collected in the 2003-2004 of National Health and Nutrition Examination Survey (NHANES). NHANES collects data from a US noninstitutionalized sample population. Participants are selected by a complex multistage, probability design which oversamples certain underrepresented subgroups to increase the generalizability of survey results. The National Center for Health Statistics research ethics review board approved all study protocols and informed consent was obtained from all survey participants.

**Figure 1:**
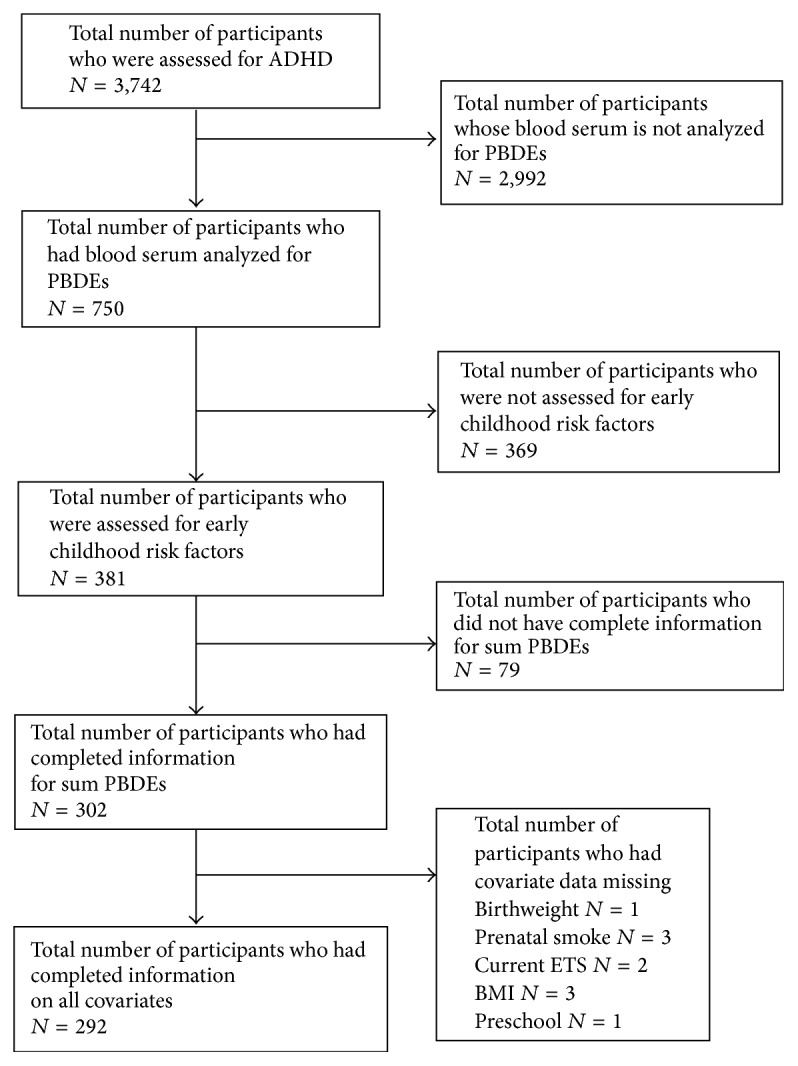
Schematic depicting derivation of sample size.

Out of the 3,742 participants aged 4 to 19 years who were asked if a health care professional told them they had ADHD, 750 individuals aged 12 to 19 had their blood serum analyzed for PBDEs. However, important early childhood risk factors for ADHD (prenatal smoke exposure and preschool attendance) were assessed only in children 0 to 15 years old which excluded children aged 16–19 (*n* = 369) which restricted this analysis to children 12 to 15 years old. Additionally, 79 individuals had missing PBDE values due to inadequate serum sample or suspected contamination of sample, leaving 302 individuals who had complete data on all covariates and PBDE measurements. One individual had information missing on both birthweight and preschool attendance, 3 individuals had information missing on both prenatal smoke exposure and BMI, and 2 individuals had missing data on current smoke exposure, resulting in 292 individuals with complete information (Figure 1).

### 2.2. Parental Reported Diagnosis of ADHD

Respondents were asked whether their child had been diagnosed with ADHD based on the question “Has a doctor or health professional ever told you that your child has attention deficit disorder (yes/no/don't know/refuse to answer)?” Refusal to answer or “don't know” responses were coded as missing. No missing data for ADHD diagnosis was present in the subpopulation used in this analysis.

### 2.3. PBDE Exposure

Serum brominated flame retardants levels were only measured in one-third of the blood samples collected in participants 12 years old and older. Detailed laboratory analysis methods are described elsewhere [13]. Briefly, samples were analyzed using automated solid-phase extraction (SPE) followed by sample clean-up and analysis by isotope dilution gas high-resolution mass spectrometry (GC-IDHRMS). We only included PBDEs in this analysis that had a detection frequency of 60% or higher (e.g., 2,2′,4-tribromodiphenyl ether (BDE-28), 2,2′,4,4′-tetrabromodiphenyl ether (BDE-47), 2,2′,4,4′,5-pentabromodiphenyl ether (BDE-99), 2,2′,4,4′,5,5′-pentabromodiphenyl ether (BDE-100), and 2,2′,4,4′,5,5′-hexabromodiphenyl ether (BDE-153)) [15]. Due to the lipophilic nature of PBDEs, we used lipid-adjusted concentrations which are more strongly correlated with the adipose tissue concentrations of the chemical [14] and on a wet weight basis.

Each PBDE congener contained left-censored data but 60% or more of the subjects had values above the limit of detection (LOD). However, due to concerns that substituting values with the LOD/√2 may lead to bias, we used multiple imputation to substitute values for left-censored chemical data [16, 17]. Briefly, PBDE concentrations falling below the LOD were coded as missing and demographic variables and the outcome variables were used in a multivariate normal model. Markov Chain Monte Carlo (MCMC) simulations were used to create 5 complete data sets with imputed values for concentrations below the LOD and estimated concentrations were drawn from these data. Note that PBDE concentrations were not imputed for the 66 individuals who had missing data due to inadequate serum sample amount or if the samples were suspected to be contaminated [15].

### 2.4. Covariates

Risk factors for ADHD identified in previous studies were considered to be potential confounders in this analysis. These included sex, age, race [18], blood lead levels [8], prenatal and current environmental tobacco smoke (ETS) exposure [8], low birthweight [19], and BMI [20]. As an indicator of socioeconomic status, we used the family poverty index ratio (PIR) which was calculated for the survey cycle year by the US Department of Health and Human Services based on self-reported household income levels. We dichotomized this variable based on standardized guidelines where participants with PIR values of 0 through 0.99 were considered below the poverty line and a PIR value of 1 and above was considered at or above poverty line. Health insurance status (yes/no) was also explored as a covariate because diagnosis of ADHD may be dependent on the ability to access health care. Preschool attendance was explored as a potential covariate because children who attend preschool exhibit different socioemotional adjustment than children who do not attend preschool [21].

### 2.5. Statistical Analysis

Stata 13 [22] was used to perform all statistical analyses using sampling weights according to the NCHS recommendations [13] to account for survey nonresponse and to produce unbiased national estimates. Analysis was also performed without weights due to small sample sizes and the possibility of undue influence by a few individuals. The weighted and unweighted prevalence of ADHD were calculated for adolescents aged 12 to 15 years who had their blood analyzed for PBDEs. The association between ADHD and potential covariates was assessed using Chi-square tests. Covariates were included in the final multivariate logistic regression model if they were associated with ADHD, a *p* value < 0.2. Additionally, lead (natural log transformed) was included as a covariate because of its strong positive association with ADHD [8, 23, 24]. Logistic regression analysis was performed using log-transformed single PBDE congeners and the sum total PBDE as a continuous exposure and categorized into tertiles. We also examined the relationship between those with the highest exposures and those with the lowest exposures by categorizing PBDEs above and below the 75th percentile. This cut-off was chosen instead of the 95th percentile which is a more commonly used extreme comparison group due to the limited sample size.

Analysis was conducted using the lipid-adjusted and wet weight PBDE concentrations.

## 3. Results

We compared select characteristics of youths 12–15 years old who had their blood analyzed for PBDEs to those of youths who did not have their blood analyzed for PBDEs to evaluate whether the included sample was representative of all youths in this age range. There were no significant differences in the sociodemographic, environmental factors or ADHD diagnosis in youth 12–15 years old who had their blood serum analyzed for PBDEs compared to youth 12–15 years old who did not have their blood serum analyzed for PBDEs (Table 1).

The weighted prevalence of parent-reported clinical ADHD diagnosis by a health care provided in this sample of youth aged 12–15 years was 13.57% (95% CI: 8.06, 21.94). Several risk factors for ADHD were identified in this sample. Prenatal ETS exposure, health insurance, attendance in preschool or daycare, and BMI were significantly associated with higher prevalence of ADHD (Table 2). However, due to the restricted sample size, there was a limited number of ADHD cases (*N* = 2) that were not covered by health insurance; therefore, health insurance was not included as a covariate in the final full model.

The geometric mean, detection frequency, and range of the individual PBDEs in youth aged 12–15 years in NHANES 2003-2004 are presented in Table 3. PBDE-28 had the lowest lipid-adjusted serum geometric mean concentration of 1.69 ng/g and a detection frequency of 84%. BDE-47 had the highest lipid-adjusted serum geometric mean concentration of 31.51 ng/g and a detection frequency of 99%. The lipid-adjusted serum geometric mean concentrations for BDE-99, BDE-100, and BDE-153 were determined to be 10.08, 6.31, and 9.28 ng/g, respectively. The detection frequency for BDE-99, BDE-100, and BDE-153 was determined to be 97% for each congener. Finally, the lipid-adjusted serum geometric concentration for the sum of all the congeners was determined to be 55.42 ng/g. Similar trends were observed for wet weight PBDE concentrations although the range of concentrations was much greater.

The bivariate relationships between PBDE exposures and parental reported ADHD are presented in Table 4. No significant associations were observed between any of the PBDE congeners (lipid-adjusted or wet weight) or sum total PBDES (lipid-adjusted or wet weight) and ADHD in the weighted or unweighted bivariate analysis. The unweighted and weighted adjusted odds ratio (AOR) after controlling for lead, sex, birthweight, BMI, daycare attendance, and prenatal smoke exposure are presented in Table 4. Similar to the bivariate logistic regression model results, there appeared to be no significant association between any of the lipid-adjusted or wet weight PBDE congeners and ADHD. There was a positive but not statistically significant association between the sum total PBDE and ADHD (weighted AOR: 1.41, 95% CI: 0.70, 2.83). Similar associations were observed for wet weight sum total PBDE and ADHD (weighted AOR: 1.24, 95% CI: 0.62, 2.50). The weighted AOR of ADHD for individuals above the 75th percentile compared to those below the 75th percentile for lipid-adjusted PBDE was 0.59 (95% CI: 0.10, 3.56) and for wet weight PBDE was 0.90 (95% CI: 0.21, 3.72). Similarly, the weighted AOR of ADHD for individuals in the highest 2nd and 3rd tertiles were 6.16 (95% CI: 1.19, 31.90) and 0.99 (95% CI: 0.23, 4.29), respectively, compared to the lowest tertile of lipid-adjusted PBDE after adjusting BMI, attendance in preschool, sex, blood lead levels, and prenatal ETS exposure. When examining the association between concentrations of ADHD using wet weight PBDE, the weighted AOR in the 2nd and 3rd tertile was 1.81 (95% CI: 0.41, 8.04) and 0.63 (95% CI: 0.17, 2.36), respectively. Similar null associations were observed for unweighted models.

## 4. Discussion

In this subpopulation of US youths, the prevalence of ADHD was 13.57% which was higher than the anticipated prevalence even when accounting for the weighting that addresses the complex survey design of NHANES. This weighted prevalence is greater than the 9.5% weighted prevalence of ADHD diagnosis among children 4 to 17 years old in 2007 from the National Survey of Children's Health [25]. However, prevalence estimates of ADHD in community samples have ranged from 2 to 18% among school aged children [26]. Similar to other population-based studies conducted in the US, we observed that prenatal ETS exposure and high BMI were associated with a higher risk of parental reported ADHD [27]. However, this cross-sectional analysis did not observe any significant association between PBDE exposure and parental report of clinical ADHD diagnosis. However, there appeared to be modestly elevated odds of parental report of clinical ADHD diagnosis among those youths with the highest sum total PBDE exposures although these associations were not statistically significant.

Animal toxicity testing [28] and studies using a human* in vitro* model [29] have suggested an association between brominated flame retardants exposure and attention behavior alterations. Further, prospective cohort studies have shown an association between increased maternal blood serum PBDE levels during pregnancy and decreased attention behaviors [11], scores on ADHD index scores [12], and educator reported odds of behavioral problems [30] in children ranging from 5 to 7 years of age. Despite the biological plausibility and epidemiological evidence of PBDE-induced neurotoxicity and epidemiological evidence of PBDE-related attention behaviors in early childhood, our cross-sectional analysis did not detect any change in odds of prevalent ADHD among those youths with the above-average exposure to PBDEs compared to youths with below-average exposure. This lack of association between PBDE exposure and ADHD may be due to several factors. First, it is possible that the mechanistic pathways in which PBDEs act on attention behaviors in animal models are different from the mechanistic pathways associated with ADHD in humans. Second, previous animal toxicity studies used a high concentration dosing regimen which may not be realistic regarding human exposure concentrations and are much higher than what was observed in this sample [31]. Third, parent recall of ADHD diagnosis may be subject to recall bias especially if the diagnosis was made when the child was very young [12]. Fourth, even though blood serum PBDEs have estimated half-life on the order of years and reflect long-term exposure, it is possible that the blood serum concentrations measured at the age of 12–15 years were not reflective of concentrations during the critical time period of ADHD development. Fifth, even though this sample was drawn from a large US based population, the concentration of PBDEs was only measured in one-third of the sample which resulted in a relatively small sample in this age group which limited our ability to categorize the data and examine individuals with the highest exposure, that is, the 95th percentile of exposure. Additionally, we were unable to account for health insurance status in our models which is an important covariate especially for an outcome based on clinical diagnosis. Finally, we did not access the restricted data from the Diagnostic Interview Schedule for Children (DISC-IV) which captures criteria that could be used to characterize mental disorders in participants following criteria specified in the Diagnostic and Statistical Manual of Mental Disorders (4th edition). Our rationale for not accessing the DISC-IV data was based on a preliminary review of the number of individuals who had completed this interview and knowing that only 1/3 of these individuals would have had their serum analyzed for PBDEs. This assessment suggested that we would have limited sample size and subsequently we did not petition to access this restricted data. Despite these many limitations, PBDEs measured in blood serum are a good biomarker of exposure and reflect an environmentally relevant concentration [31]. Additionally, this study utilized parental recall of a physician diagnosis as opposed to a parent or educator diagnosis, which strengthened the validity of the outcome. We were also able to control for many important confounders.

## 5. Conclusions

Youths aged 12 to 15 years residing in the United States are exposed to several brominated flame retardants. While this cross-sectional study did not support an association between brominated flame retardants and ADHD in these youth 12 to 15 years old, previous research has provided some evidence that* in utero* exposure to PBDEs is a risk factor for attention behavior disorders in younger children. Subsequently, more research is needed to determine if there is a critical window during development for PBDE toxicity or if PBDE exposure remains a risk factor for ADHD as children get older.

## Figures and Tables

**Table 1:** Percentage of sociodemographic and environmental factors and ADHD diagnosis for youth 12–15 years old.

	All youth 12–15 years old (*N* = 1,131)	Those who had the blood analyzed for PBDEs (*N* = 381)	Those who did not have their blood analyzed for PBDEs (*N* = 750)	Chi-square value (*p* value)
Age (years)				1.09 (0.30)
12-13	582 (51.47)	202 (53.24)	373 (50.63)	
14-15	549 (48.53)	179 (46.76)	377 (49.38)	
Missing	0	0	0	

Sex				2.70 (0.10)
Male	582 (51.26)	183 (47.35)	399 (53.44)	
Female	549 (48.74)	198 (52.65)	351 (46.56)	
Missing	0	0	0	

Race				2.57 (0.46)
Mexican American	350 (31.75)	127 (34.12)	223 (30.63)	
Other, including other Hispanic and multiracial	76 (7.08)	23 (5.88)	53 (7.50)	
Non-Hispanic white	292 (25.08)	90 (23.82)	202 (26.09)	
Non-Hispanic black	413 (36.10)	141 (36.17)	272 (35.78)	
Missing	0	0	0	

PIR				1.21 (0.27)
0–0.99	332 (32.15)	120 (34.44)	212 (30.93)	
≥1	746 (67.85)	244 (65.56)	502 (69.07)	
Missing	53	17	36	

Prenatal ETS^a^ exposure				0.22 (0.64)
Yes	191 (16.84)	67 (17.65)	124 (16.41)	
No	927 (83.16)	309 (82.35)	618 (83.59)	
Missing	13	5	8	

Current ETS^a^ exposure in home				0.01 (0.94)
Yes	253 (22.55)	86 (23.53)	167 (22.03)	
No	863 (77.45)	291 (76.47)	572 (77.97)	
Missing	15	4	11	

Covered by health insurance				0.04 (0.84)
Yes	954 (85.44)	322 (85.29)	632 (85.47)	
No	159 (14.56)	55 (14.71)	104 (14.53)	
Missing	18	4	14	

Birthweight				0.26 (0.61)
≥2,500 g	1,000 (88.57)	334 (87.94)	666 (88.91)	
<2,500 g	129 (11.43)	46 (12.06)	83 (14.53)	
Missing	2	1	1	

Attended preschool/daycare				2.40 (0.12)
Yes	775 (69.29)	249(66.47)	526 (70.78)	
No	353 (30.71)	130 (33.53)	223 (29.22)	
Missing	3	2	1	

Body Mass Index (BMI)				2.01 (0.37)
Underweight/normal	676 (61.33)	244 (64.12)	432 (59.84)	
Overweight	250 (23.16)	80 (21.18)	170 (24.22)	
Obese	170 (15.51)	54 (14.71)	116 (15.94)	
Missing	35	3	32	

Blood lead quartiles				3.41 (0.33)
1st quartile	418 (41.22)	156 (44.71)	262 (39.38)	
2nd quartile	309 (30.51)	106 (30.00)	203 (30.78)	
3rd quartile	180 (17.86)	57 (16.47)	123 (18.59)	
4th quartile	16 (10.41)	31 (8.82)	75 (11.25)	
Missing	0	31	87	

ADHD diagnosis				0.43 (0.51)
Yes	113 (9.22)	35 (7.55)	78 (10.11)	
No	1,016 (90.77)	346 (92.45)	670 (89.89)	
Missing	2	0	2	

^a^Environmental Tobacco Smoke.

**Table 2:** Prevalence of ADHD diagnosis by a health care professional among youth 12–15 years old in NHANES 2003-2004.

Variable	Number of children with ADHD	Number of children without ADHD	Weighted percent with ADHD (95% CI)	*p* value^a^
Total	35	346	13.57 (8.06, 21.94)	

Age (years)				0.48
12-13	23	179	8.37 (3.95, 16.88)	
14-15	12	167	5.20 (3.21, 8.31)	

Sex				0.10
Female	9	189	4.12 (1.33, 12.05)	
Male	26	157	9.45 (5.58, 15.56)	

Race				0.21
Mexican American	6	121	0.69 (0.21, 2.21)	
Other, including other Hispanic and multiracial	2	21	1.25 (0.26, 5.69)	
Non-Hispanic white	16	74	10.44 (5.56, 18.78)	
Non-Hispanic black	11	130	1.19 (0.69, 2.06)	

PIR^b^				0.54
0–0.99	5	115	2.37 (0.74, 7.28)	
≥1	29	215	10.80 (5.94, 18.85)	
Missing	1	16		

Prenatal ETS^c^ exposure				0.04
Yes	21	288	4.66 (2.24, 9.44)	
No	12	55	7.57 (4.71, 11.96)	
Missing	2	3		

Current ETS^c^ exposure in home				0.50
Yes	25	266	4.22 (1.94, 8.92)	
No	10	76	9.40 (5.39, 15.88)	
Missing	0	4		

Covered by health insurance				<0.001
Yes	33	289	13.40 (7.89, 21.83)	
No	2	53	0.22 (0.19, 0.26)	
Missing	0	4		

Birthweight				0.08
≥2,500 g	27	307	10.35 (5.34, 19.10)	
<2,500 g	8	38	3.23 (1.60, 6.40)	
Missing	0	1		

Attended preschool/daycare				0.02
Yes	29	220	11.39 (6.81, 18.45)	
No	5	125	2.08 (0.65, 6.49)	
Missing	1	1		

Body Mass Index (BMI)				0.05
Underweight/normal	20	224	5.87 (3.46, 9.79)	
Overweight	7	73	3.65 (1.45, 8.89)	
Obese	8	46	4.09 (1.44, 11.07)	
Missing	0	3		

Blood lead quartiles				0.49
1st quartile	10	146	4.43 (1.73, 10.87)	
2nd quartile	10	96	3.28 (1.24, 8.37)	
3rd quartile	4	53	2.24 (0.71, 6.82)	
4th quartile	3	28	0.82 (0.15, 4.39)	
Missing	8	23		

^a^ *p* value for Chi-square analysis, ^b^Poverty Index Ratio, and ^c^Environmental Tobacco Smoke.

**Table 3:** Geometric mean, percent above LOD, and range of lipid-adjusted and non-lipid-adjusted PBDEs in youth aged 12–15 years after imputation for values below LOD (NHANES 2003-2004).

Lipid-adjusted (ng/g)			Non-lipid-adjusted (pg/g)		
PBDE congener	Geometric mean (95% CI)	Range	Geometric mean (95% CI)	Range	% above LOD
BDE-28	1.69 (1.55, 1.84)	0.30–14	7.11 (6.52, 7.75)	0.86–77	84
BDE-47	31.51 (28.39, 34.98)	3–488	152.63 (137.46,169.48)	10–2291	99
BDE-99	10.08 (9.07, 11.21)	0.45–131	37.33 (33.40, 41.72)	2–738	97
BDE-100	6.31 (5.69, 7.00)	0.51–137	29.57 (26.63, 32.82)	2–644	97
BDE-153	9.28 (8.35, 10.31)	0.80–188	43.69 (39.24, 48.63)	2–880	97

Sum total PBDE	55.42 (49.84, 61.63)	5–884	282.15 (255.45, 311.64)	15–4146	NA

**Table 4:** Crude and adjusted odds ratio (OR) for ADHD diagnosed by a health care provider and PBDE exposure.

	ADHD Yes	ADHD No	Weighted crude odds^b^ (95% CI)	*p* value	Unweighted crude odds (95% CI)	*p* value	Weighted adjusted odds^bc^ (95% CI)	*p* value	Unweighted adjusted odds^bc^ (95% CI)	*p* values
BDE-28^a^										
Lipid-adjusted	24	282	1.01 (0.51, 1.99)	0.98	1.11 (0.63, 1.94)	0.72	1.16 (0.51, 2.67)	0.68	0.97 (0.52, 1.82)	0.93
Wet weight	24	282	0.93 (0.42, 2.08)	0.84	0.90 (0.51, 1.57)	0.70	0.97 (0.38, 2.45)	0.94	0.85 (0.50, 1.78)	0.86

BDE-47^a^										
Lipid-adjusted	25	284	0.83 (0.48, 1.44)	0.52	0.99 (0.64, 1.54)	0.97	1.36 (0.72, 2.56)	0.32	1.03 (0.63, 1.67)	0.91
Wet weight	25	284	1.17 (0.68, 2.05)	0.54	1.01 (0.65, 1.57)	0.96	1.22 (0.62, 2.40)	0.55	1.01 (0.62, 1.66)	0.96

BDE-99^a^										
Lipid-adjusted	24	279	0.82 (0.44, 1.52)	0.50	0.97 (0.61, 1.46)	0.80	1.51 (0.70, 3.25)	0.26	1.06 (0.65, 1.71)	0.82
Wet weight	24	279	1.25 (0.65, 2.42)	0.47	1.06 (0.69, 1.63)	0.79	1.37 (0.63, 2.98)	0.39	1.04 (0.64, 1.69)	0.87

BDE-100^a^										
Lipid-adjusted	25	288	0.72 (0.36, 1.44)	0.32	0.95 (0.61, 1.48)	0.83	1.53 (0.73, 3.23)	0.24	1.04 (0.64, 1.69)	0.84
Wet weight	25	288	1.36 (0.70, 2.64)	0.34	1.05 (0.68, 1.63)	0.82	1.37 (0.66, 2.82)	0.37	1.03 (0.64, 1.67)	0.91

BDE-153^a^										
Lipid-adjusted	25	288	1.27 (0.56, 2.90)	0.54	1.02 (0.66, 1.57)	0.93	1.43 (0.57, 3.56)	0.42	1.02 (0.61, 1.65)	0.95
Wet weight	25	288	1.29 (0.52, 3.21)	0.56	0.98 (0.64, 1.51)	0.93	0.27 (0.56, 2.90)	0.54	1.00 (0.60, 1.67)	0.99

Sum PBDEs^a^										
Lipid-adjusted	24	278	1.24 (0.63,2.42)	0.50	0.99 (0.62, 1.58)	0.97	1.41 (0.70, 2.83)	0.31	0.99 (0.60, 1.64)	0.97
Wet weight	24	278	1.19 (0.63, 2.25)	0.56	1.00 (0.63, 1.59)	0.99	1.24 (0.62, 2.50)	0.52	0.99 (0.58, 1.68)	0.96

Sum PBDEs										
Lipid-adjusted below 75%	11	139	Ref	Ref	Ref	Ref	Ref	Ref	Ref	Ref
Above 75%	13	139	0.87 (0.19, 3.91)	0.84	0.76 (0.31, 1.84)	0.54	0.59 (0.10, 3.56)	0.53	0.70 (0.38, 1.29)	0.26
Wet weight										
Below 75%	16	205	Ref		Ref		Ref	Ref	Ref	Ref
≥75%	8	73	1.03 (0.19, 5.65)	0.97	0.72 (0.29, 1.75)	0.47	0.90 (0.21, 3.72)	0.87	0.60 (0.22, 1.64)	0.32

Sum PBDE										
Lipid-adjusted 1st tertile (lowest)	11	131	Ref	Ref	Ref	Ref	Ref	Ref	Ref	Ref
2nd tertile	5	78	5.75 (2.02, 16.34)	0.003	1.31 (0.44, 3.92)	0.63	6.16 (1.19, 31.90)	0.03	1.34 (0.39, 4.64)	0.64
3rd tertile (highest)	8	69	1.09 (0.23, 5.22) 1.64 (0.56, 4.80)	0.91	0.73 (0.28, 1.90)	0.52	0.99 (0.23, 4.29)	0.99	0.66 (0.23, 1.94)	0.45
Wet weight										
1st tertile (lowest)	10	133	Ref		Ref		Ref		Ref	Ref
2nd tertile	5	77	1.55 (0.39, 6.28)	0.51	1.23 (0.33, 4.63)	0.76	1.81 (0.41, 8.04)	0.41	1.17 (0.33, 4.16)	0.81
3rd tertile (highest)	9	68	1.96 (0.66, 5.81)	0.21	1.17 (0.37, 3.76)	0.79	0.63 (0.17, 2.36)	0.46	0.47 (0.16, 1.38)	0.17

^a^Natural log transformed.

^b^Weighted analysis was conducted using NHANES sampling weights to account for the complex sampling design. According to NHANES guidelines, the sample weights were used which corresponded to subsample that had their serum analyzed for PBDEs [15].

^c^Adjusted for lead, sex, birthweight, BMI, daycare attendance, and prenatal smoke exposure.

## List of Abbreviations

ADHD: Attention Deficient Hyperactivity Disorder
BDE-28: 2, 2′,4-Tribromodiphenyl ether (BDE-28)
BDE-47: 2,2′,4,4′-Tetrabromodiphenyl ether
BDE-99: 2,2′,4,4′,5-Pentabromodiphenyl
BDE-100: 2,2′,4,4′,5,5′-Pentabromodiphenyl ether
BDE-153: 2,2′,4,4′,5,5′-Hexabromodiphenyl ether
BMI: Body Mass Index
DISC-IV: Diagnostic Interview for Children
DSM: Diagnostic and Statistical Manual of Mental Disorders
PBDE: Polybrominated diphenyl ether
PBB: Polybrominated biphenyl
CHAMACOS: Center for Health Assessment of Mothers and Children of Salinas
LOD: Limit of detection
NHANES: National Health and Nutrition Examination Survey
NSCH: National Survey of Children's Health.
